# Advantages of functional single-cell isolation method over standard agar plate dilution method as a tool for studying denitrifying bacteria in rice paddy soil

**DOI:** 10.1186/2191-0855-2-50

**Published:** 2012-09-18

**Authors:** Tomoyasu Nishizawa, Kanako Tago, Yusuke Uei, Satoshi Ishii, Kazuo Isobe, Shigeto Otsuka, Keishi Senoo

**Affiliations:** 1 Department of Applied Biological Chemistry, Graduate School of Agricultural and Life Sciences, The University of Tokyo, 1-1-1 Yayoi, Bunkyo-ku, Tokyo, 113-8657, Japan; 2 Environmental Biofunction Division, National Institute for Agro-Environmental Sciences, 3-1-3 Kannondai, Tsukuba, Ibaraki, 305-8604, Japan; 3 Present address: Division of Environmental Engineering, Hokkaido University, Sapporo, Hokkaido, 060-8628, Japan

**Keywords:** 16S rRNA gene, Denitrifying bacteria, Functional single-cell isolation, Phylogenetic analysis, Rice paddy soil

## Abstract

We recently established a method for isolating functional single cells from environmental samples using a micromanipulator (Functional single-cell (FSC) isolation), and applied it to the study of denitrifying bacteria in rice paddy soil (Ashida et al. 2010. Appl Microbiol Biotechnol 85:1211–1217). To further examine the advantages and possible disadvantages of the FSC method, we isolated denitrifying bacteria from the same rice paddy soil sample using both FSC and standard agar plate dilution (APD) methods and compared in this study. The proportion of denitrifying bacteria in the total isolates was more than 6-fold larger with FSC isolation (57.1%) compared with the APD method (9.2%). Denitrifying bacteria belonging to Alphaproteobacteria and Bacilli were commonly isolated using both methods, whereas those belonging to Betaproteobacteria, which had been found to be active in the denitrification-inductive paddy soil, were isolated only with the FSC method. On the other hand, Actinobacteria were only isolated using the APD method. The mean potential denitrification activity of the FSC isolates was higher than that of the APD isolates. Overall, FSC isolation was confirmed to be an excellent method for studying denitrifying bacteria compared with the standard agar plate dilution method.

## Introduction

Denitrification is a microbial respiratory process where nitrate/nitrite are reduced in a stepwise manner to form the gaseous end products NO, N_2_O, and N_2_ ([Zumft 1997]). A variety of microorganisms, including some archaea and fungi, are known to have denitrification capabilities ([Hayatsu et al. 2008]). Denitrification is an important process during the nitrogen transformation of arable soils, because it causes N_2_O emission in upland soils and nitrogen loss in rice paddy soils ([Ishii et al. 2011]). Denitrification of rice paddy soil was reported as early as 1907 ([Ishii et al. 2011]), however, the microbial populations responsible for denitrification have not been investigated for a long period.

To overcome this situation, we recently established a method for isolating functional single cells from environments using a micromanipulator (Functional single-cell (FSC) isolation), which we applied in studying denitrifying bacteria in rice paddy soil ([Ashida et al. 2010]). FSC isolation was used to isolate denitrifying bacteria from various rice paddy and rice-soybean rotation fields and their phylogenetic and functional diversity were examined ([Tago et al. 2011]). Oligotrophic denitrifying bacteria carrying previously uncharacterized functional gene sequences were also isolated using the FSC method ([Ishii et al. 2010a]). These studies clearly indicated the usefulness of the FSC isolation method as a tool for studying denitrifying bacteria in soil. However, the advantages and possible disadvantages of the FSC isolation method compared with the standard isolation method have not been well understood.

In this study, we isolated denitrifying bacteria from the same soil sample using both the FSC method and the standard agar plate dilution method. The following criteria were evaluated for each method and compared: (1) acquisition rate of denitrifying bacteria, *i.e*., the final number of denitrifying bacteria over the number of well-isolated candidate single colonies during the isolation process; (2) taxonomic composition; and (3) potential denitrification activity of the obtained denitrifying isolates.

## Materials and methods

### Soil samples

Soil samples were collected from the upper 10 cm soil layer of a rice paddy field at Niigata Agricultural Research Institute (37°20′N, 138°45′E), Japan, on April 7 (before the cultivation period) in 2009. Ten soil samples were randomly collected from the field, which were combined together and sieved through a 2-mm mesh, and stored at 4°C until use. The soil type is gley soil, and its physicochemical properties are as follows: pH (H_2_O): 5.6; total carbon: 14.0 g kg^-1^ dry soil; and total nitrogen: 1.3 g kg^-1^ dry soil.

### Soil microcosm and isolation

To perform functional single-cell isolation of denitrifying bacteria, a denitrification-inductive soil microcosm was set up according to the method of [Tago et al. (2011]), with some modifications. In brief, 1.0 g of the soil sample was placed in a 10 ml glass serum vial (Nichiden-Rika Glass, Kobe, Japan), which was submerged in sterile MilliQ water (3 ml) for 1 week at 30°C (pre-incubation). A 2.5 ml layer of clear water was removed, and 0.05 mg-C succinate (1.4 μmol) and 0.01 mg-N nitrate (2.1 μmol) were added as an electron donor and acceptor for denitrification, respectively. Nalidixic acid (final concentration, 20 μg g^−1^ dry soil), pilomidic acid (10 μg g^−1^ dry soil), and pipemidic acid (20 μg g^-1^ dry soil) were also added to the soil as bacterial cell division inhibitors. The vial was sealed with a butyl rubber stopper and the headspace air was replaced with Ar-C_2_H_2_ (90:10) gas. The prepared soil microcosm was incubated at 30°C for 16 h to elongate bacterial cell ready to grow by succinate assimilation under denitrification-inductive conditions ([Tago et al. 2011]).

After live staining with 5-carboxyfluorescein diacetate-acetoxymethyl ester (CFDA-AM), single elongated cells were individually captured using a micromanipulator under a fluorescent microscope (Functional single-cell isolation, [Ashida et al. 2010]), and transferred into separate 10 ml vials containing 100-fold diluted nutrient broth medium supplemented with 0.3 mM sodium nitrate and 4.4 mM sodium succinate (DNB-NS) liquid medium. After anaerobic cultivation at 30°C for 2 weeks, each cell suspension was streaked onto DNB-NS agar medium and incubated anaerobically at 30°C for 2 weeks to obtain well-isolated single colonies ([Tago et al. 2011]). Denitrifying bacteria was isolated from the incubated soil using the standard agar plate dilution (APD) method. An aliquot (100 Âµl) of the diluted soil suspension was spread onto DNB-NS agar, and incubated anaerobically at 30Â°C for 2 weeks to obtain single colonies.

### Measurement of potential activity of denitrification

The potential activities of denitrification, proportion (%) of nitrate reduced to N_2_O in two weeks, of denitrifying bacteria obtained using the FSC and APD methods were determined with the acetylene-block method ([Tiedje 1994]), as described previously ([Tago et al. 2011]). The vials containing DNB-NS medium were inoculated with each strain, and the head-space air was replaced with Ar-C_2_H_2_ (90:10) gas. After incubation at 30°C for 2 weeks, a portion (0.5 ml) of the head-space gas was analyzed for N_2_O by gas chromatography ([Saito et al. 2008]). The quantity of water-dissolved N_2_O gas was calculated as the Bunsen absorption coefficient ([Tiedje 1994]). Strains reducing more than 20% of the added nitrate to N_2_O were considered to be denitrifying bacteria in this study ([Tiedje 1994]; [Tago et al. 2011]). A two-sided, unpaired Student’s *t*-test was performed to statistically analyze the denitrifying activity.

### DNA extraction and PCR amplification

DNA was extracted from single colonies as described previously ([Ashida et al. 2010]), and used for PCR amplification. The bacterial 16S rRNA gene was amplified using the m-27 F (5′-AGRGTTTGATYMTGGCTCAG-3′) primer and the m-1492R (5′-GGYTACCTTGTTACGACTT-3′) primer pair ([Tyson et al. 2004]), as described previously ([Tago et al. 2011]).

### Amplified ribosomal DNA restriction analysis (ARDRA)-based profiling

The PCR amplicons of 16S rRNA gene were purified with a Wizard SV Gel and PCR Clean-Up System (Promega, Madison, WI, USA) and condensed to a final volume of 20 μl in sterilized-MilliQ water. Aliquots (3 μl) of the DNA samples were individually digested with HaeIII (Toyobo, Tokyo, Japan) and RsaI (Takara Bio), according to the manufacturer’s instructions. To determine the precise length of generated DNA fragments, the digested DNA samples were subjected to MetaPhor Agarose (Takara Bio) gel (3%) electrophoresis. Grouping of the denitrifying isolates was performed based on the ARDRA gel profiles.

### Sequencing and phylogenetic analysis

The 16S rRNA gene PCR amplicons derived from one to three representative strains from each ARDRA group, *i.e*., a total of 47 strains, were purified and directly sequenced as described previously ([Ashida et al. 2010]). DNA sequencing reactions were performed using a BigDye™ Terminator Cycle Sequencing Ready Reaction Kit (Applied Biosystems), according to the manufacturer’s protocol, and analyzed using a PE Applied Biosystems Automated DNA Sequencer (model 3130*xl*). Based on the gene sequences, taxonomic assignments of the strains at the genus level were performed using RDP Naïve Bayesian classifier, with an 80% bootstrap cutoff ([Cole et al. 2009]). Multiple alignments were performed using CLUSTAL W (ver. 1.83) ([Thompson et al. 1994]). Phylogenetic trees were constructed using the neighbor-joining method with Kimura-2 parameters and 500 bootstrap replicates, in the MEGA4 program ([Tamura et al. 2007]).

### Accession numbers of nucleotide sequences

The sequence data of the bacterial 16S rRNA gene produced in this study have been deposited in the DDBJ database under accession numbers AB696837 to AB696882.

## Results

### Isolation of denitrifying bacteria

FSC isolation captured 90 elongated cells and after cultivation 77 well-isolated single colonies were obtained from the soil sample. Of these, 44 strains reduced more than 20% of nitrate in the DNB-NS liquid medium to N_2_O and were considered as denitrifying bacteria ([Tago et al. 2011]). The acquisition rate of denitrifying bacteria was calculated as the number of denitrifying bacteria obtained over the number of well-isolated single colonies, which was 57.1% (Table 1). We obtained 357 well-isolated single colonies using this agar plate dilution (APD) isolation method. Of these, 33 strains were considered to be denitrifying bacteria. The acquisition rate of denitrifying bacteria was 9.2%.

**Table 1:** Acquisition rate of denitrifying bacteria using the FSC and APD methods

**Method**^**a**^	**number of well-isolated single colonies**	**number of isolates showing NO**_**3**_^**−**^**reduction**		**mean of potential denitrifying activity ± SD,%**^**c**^	**Acquisition rate of denitrifying bacteria (%)**
		**<20%**	**≥20%**^**b**^		
FSC	77	7	44	53.1 ± 21.2	57.1
APD	357	10	33	36.8 ± 16.1	9.2

^a^FSC: Functional single-cell isolation method; APD: agar plate dilution method.

^b^regarded as denitrifying bacteria.

^c^Proportion (%) of nitrate reduced to N_2_O in two weeks.

### 16S rRNA gene-based phylogenetic analysis of denitrifying bacteria

The rough grouping of the denitrifying strains obtained using the FSC and APD isolation methods were performed based on ARDRA. According to the ARDRA gel profiles, 44 (FSC) and 33 (APD) strains were classified into 17 and 12 restriction fragment length polymorphism (RFLP) types, respectively. Their taxonomic positions were analyzed based on their 16S rRNA gene sequences (Table 2). Strains isolated by the FSC isolation method were classified into the classes Alphaproteobacteria, Betaproteobacteria, and Bacilli. Strains isolated by the APD isolation method were classified into the classes Alphaproteobacteria, Actinobacteria, and Bacilli. Strains belonging to the genera *Bradyrhizobium* (class Alphaproteobacteria) and *Bacillus* (class Bacilli) were commonly obtained using both the FSC and APD isolation methods. Among the Betaproteobacteria in the FSC isolates, bacteria belonging to the order Rhodocyclales, especially *Zoogloea* strains, were most frequently obtained. Within the Actinobacteria of the APD isolates, bacteria belonging to the order Actinomycetales were obtained.

**Table 2:** Taxonomic composition of denitrifying bacteria and their denitrifying activity

**Taxon**	**Genus**	**Number of isolates (mean of potential denitrifying activity ± SD,%)**^**a**^	
**Class and Order**		**FSC**	**APD**
**Alphaproteobacteria**			
Rhizobiales	*Bradyrhizobium*	12 (53.4 ± 18.7)	11 (52.0 ± 15.9)
Rhodospirillales	*Azospirillum*	1 (69.3)	
total		13 (54.6 ± 18.4)	
**Betaproteobacteria**			
Burkholderiales	*Ralstonia*	3 (33.7 ± 18.2)	
	*Curvibacter*	1 (43.1)	
	*Ideonella*	3 (48.5 ± 19.1)	
Neisseriales	*Pseudogulbenkiania*	3 (60.8 ± 8.6)	
Rhodocyclales	*Azoarcus*	1 (50.4)	
	*Dechloromonas*	1 (23.4)	
	*Zoogloea*	14 (63.1 ± 23.2)	
	Unclassified Rhodocyclales	3 (50.9 ± 23.3)	
total		29 (54.5 ± 21.8)	
**Actinobacteria**			
Actinomycetales	*Microbacterium*		1 (32.1)
	*Arthrobacter*		1 (37.0)
	*Micromonospora*		1 (25.9)
	*Marmoricola*		1 (20.6)
	*Streptomyces*		4 (27.7 ± 10.4)
total			8 (28.3 ± 8.3)
**Bacilli**			
Bacillales	*Bacillus*	2 (22.7)	14 (29.7 ± 10.4)

^a^Proportion (%) of nitrate reduced to N_2_O in two weeks.

**Figure 1:**
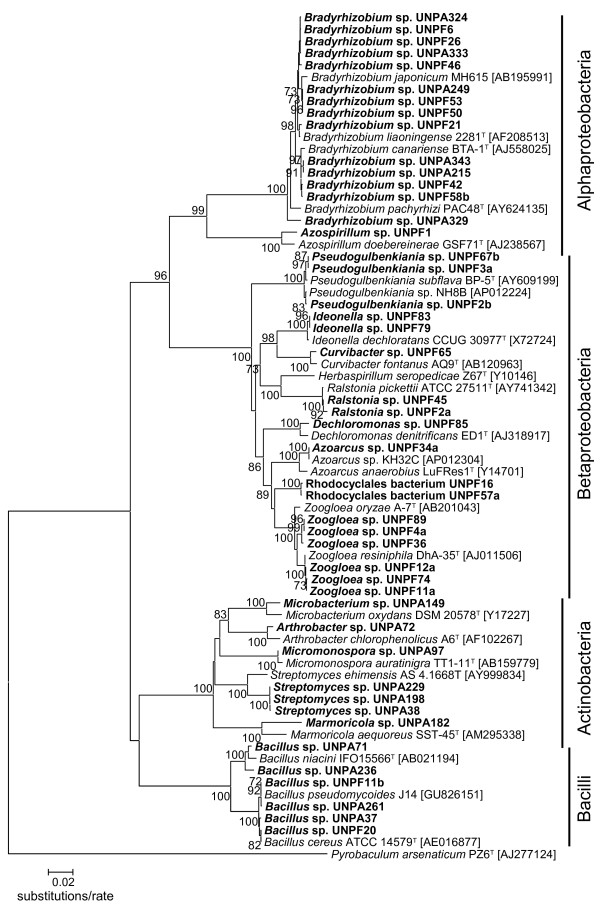
**Phylogenetic tree based on the 16S rRNA gene sequences of denitrifying bacteria obtained using the FSC and APD isolation methods.** Scale bar indicates the substitutions per site. Bootstrap values at the nodes are the percentages of 500 bootstrap replicates. Values more than 70% are indicated at branches. Bold letters indicate the sequence obtained in this study. Accession numbers are shown in parentheses. UNPF: FSC isolates; UNPA: APD isolates.

Figure 1 shows phylogenetic relationships based on the 16S rRNA gene sequences of 30 and 17 strains obtained using the FSC isolation method and the APD isolation method based on the RFLP types, respectively. A phylogenetically close relationship was found among *Bradyrhizobium* strains and among *Bacillus* strains, both of which were commonly isolated using both isolation methods. Three unique clusters were found in the phylogenetic tree, *i.e.,* one formed by *Zoogloea* strains (UNPF89, UNPF4a and UNPF36) from the FSC isolates, one formed by Rhodocyclales bacterium strains (UNPF16 and UNPF57a) from the FSC isolates, and another by *Streptomyces* strains (UNPA229, UNPA198, and UNPA38) from the APD isolates.

### Potential activity of denitrification

**Figure 2:**
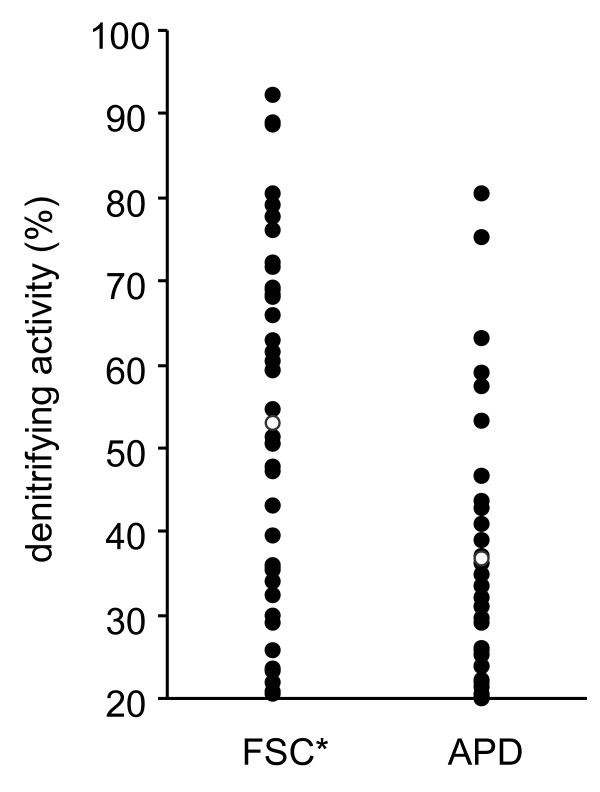
**Denitrifying activity of isolates obtained using the FSC and APD isolation methods.** Open circles indicate the average of the activities. FSC: Functional single-cell isolation; APD: agar plate dilution method. *, P < 0.01.

The mean potential activity of denitrification, proportion (%) of nitrate reduced to N_2_O in two weeks, of the FSC isolates was 53.1%, ranging from 20% to 92%, whereas that of the APD isolates was 36.8%, ranging from 20% to 81% (Figure 2). The mean potential activity of denitrification of the strains belonging to each class and genus is shown in Table 2. The mean potential activity of denitrification of the strains obtained by FSC isolation (53.1%) was significantly higher (*P* < 0.01) than those obtained by APD isolation (36.8%) (Figure 2).

## Discussion

This study isolated denitrifying bacteria from the same rice paddy soil sample using both FSC and APD isolation methods. The overall proportion of denitrifying bacteria isolates was more than 6-fold in the FSC isolation (57.1%; 44/77) compared with APD isolation (9.2%; 33/357). A similar large proportion (45%; 37/82) was also observed in our previous FSC isolation of denitrifying bacteria from another type of rice paddy soil. APD isolation resulted in a low proportion, although the DNB-NS medium under an anaerobic condition was used to grow denitrifying bacteria preferentially. FSC isolation was confirmed as a highly efficient method for obtaining denitrifying bacteria from soil, compared with the standard agar-plating method. Strains belonging to the genera *Bradyrhizobium* (class Alphaproteobacteria) were frequently isolated by both FSC (12 strains) and APD (11 strains) isolation methods, suggesting that *Bradyrhizobium* is one of the abundant denitrifying bacteria present in the Niigata paddy soil.

The taxonomic composition of the remaining strains was remarkably different in the FSC isolates and APD isolates. One striking difference was that strains belonging to Betaproteobacteria were only isolated in the FSC isolation method. This is probably because Betaproteobacteria bacteria were outcompeted by faster-growing microorganisms on DNB-NS agar plates in APD isolation. In contrast, FSC isolation provides a habitat where there is no competition for the bacterial growth substrate ([Ishii et al. 2010b]), so a number of Betaproteobacteria denitrifiers (27 strains) were isolated by the FSC isolation method in this study. Strains belonging to *Zoogloea* in Betaproteobacteria were most frequently isolated (14 strains), suggesting that *Zoogloea* is another abundant denitrifying bacteria in the Niigata paddy soil. In our soil RNA-based culture-independent study, Betaproteobacteria including *Zoogloea* was found to be active in the denitrification-inductive microcosm of the Niigata paddy soil ([Yoshida et al. 2010]). The FSC isolation method seems good to isolate this type of active, but relatively slow-growing denitrifying bacteria in soil.

Another striking difference was that strains belonging to Actinobacteria were isolated only by the APD isolation method. It was possible that the cell division inhibitors (nalidixic acid, pilomidic acid, and pipemidic acid) used for FSC isolation were not very effective in elongating Actinobacteria cells. In fact, nalidixic acid-resistant actinomycetes are known (Kieser et al. [2000]). In addition, some actinomycete strains also possess a pathway for the metabolism of pilomidic acid ([Hironaka and Nishikawa 1975]). Other suitable cell division inhibitors should be sought in order to isolate Actinobacteria denitrifiers by the FSC isolation method. On the other hand, strains belonging to *Bacillus* were isolated less frequently using the FSC isolation method (2 strains) than the APD isolation method (14 strains). This means that some population of *Bacillus* denitrifier could not form elongated cells under the condition used in this study, but the reason is unclear at present.

Both methods isolated strains with potential activity of denitrification ranging from 20% to over 80%, but the mean denitrification activity of the FSC isolates was higher (53.1%) than that of the APD isolates (36.8%). This was largely because of the relatively high denitrification activities of Betaproteobacteria strains isolated only in the FSC method, and the low activities of Actinobacteria strains isolated only in the APD method.

In conclusions, our current study indicated that the FSC isolation is an excellent method to isolate active denitrifying bacteria from rice paddy soil with high efficiency. However, the current FSC method was not always the best, because some taxonomic groups (*e.g.,* Actinobacteria) were rarely isolated in the FSC method.

## Competing interest

The authors declare that they have no competing interests.

## References

[B1] Ashida N Ishii S Hayano S Tago K Tsuji T Yoshimura Y Otsuka S Senoo K Isolation of functional single cells from environments using a micromanipulator: application to study denitrifying bacteria Appl Microbiol Biotechnol 2010 85 1211 1217 10.1007/s00253-009-2330-z 19936739

[B2] Cole JR Wang Q Cardenas E Fish J Chai B Farris RJ Kulam-Syed-Mohideen AS McGarrell DM Marsh T Garrity GM Tiedje JM The Ribosomal Database Project: improved alignments and new tools for rRNA analysis Nucleic Acids Res 2009 37 D141 D145 10.1093/nar/gkn879 19004872 PMC2686447

[B3] Hayatsu M Tago K Saito M Various players in the nitrogen cycle: Diversity and functions of the microorganisms involved in nitrification and denitrification Soil Sci Plant Nutr 2008 54 33 45 10.1111/j.1747-0765.2007.00195.x

[B4] Hironaka J Nishikawa S A new antibacterial metabolite converted from piromidic acid by *Streptomyces* sp J Ferment Technol 1975 53 372 379

[B5] Ishii S Tago K Senoo K Single-cell analysis and isolation for microbiology and biotechnology: methods and applications Appl Microbiol Biotechnol 2010 86 1281 1292 10.1007/s00253-010-2524-4 20309540

[B6] Ishii S Yamamoto M Tago K Otsuka S Senoo K Microbial populations in various paddy soils respond differently to denitrification-inducing conditions, albeit background bacterial populations are similar Soil Sci Plant Nutr 2010 56 220 224 10.1111/j.1747-0765.2010.00453.x

[B7] Ishii S Ikeda S Minamisawa K Senoo K Nitrogen cycling in rice paddy environments: past achievements and future challenges Microbes Environ 2011 26 282 292 10.1264/jsme2.ME11293 22008507

[B8] Kieser T Bibb MJ Buttner MJ Chater KF Hopwood DA Practical Streptomyces Genetics 2000 2 John Innes Foundation, England, Norwich

[B9] Saito T Ishii S Otsuka S Nishiyama M Senoo K Identification of novel Betaproteobacteria in a succinate-assimilating population in denitrifying rice paddy soil by using stable isotope probing Microbes Environ 2008 23 192 200 10.1264/jsme2.23.192 21558708

[B10] Tago K Ishii S Nishizawa T Otsuka S Senoo K Phylogenetic and functional diversity of denitrifying bacteria isolated from various rice paddy and rice-soybean rotation fields Microbes Environ 2011 26 30 35 10.1264/jsme2.ME10167 21487200

[B11] Tamura K Dudley J Nei M Kumar S MEGA4: molecular evolutionary genetics analysis (MEGA) software version 4.0 Mol Biol Evol 2007 24 1596 1599 10.1093/molbev/msm092 17488738

[B12] Thompson JD Desmond GH Gibson TJ CLUSTAL W: improving the sensitivity of progressive multiple sequence alignment through sequence weighting, position-specific gap penalties and weight matrix choice Nucleic Acids Res 1994 22 4673 4680 10.1093/nar/22.22.4673 7984417 PMC308517

[B13] Tiedje JM Weaver RW, Angle JS, Bottomley PJ Denitrifiers Methods of soil analysis, Part 2: Microbiological and biochemical properties 1994 Soil Science Society of America, Madison 245 267

[B14] Tyson GW Chapman J Hugenholtz P Allen EE Ram RJ Richardson PM Solovyev VV Rubin EM Rokhsar DS Banfield JF Community structure and metabolism through reconstruction of microbial genomes from the environment Nature 2004 428 37 43 10.1038/nature02340 14961025

[B15] Yoshida M Ishii S Otsuka S Senoo K Expressions of denitrification functional genes in rice paddy soil Conference proceedings of the 13th International Society for Microbial Ecology, PS.22.061 2010 814

[B16] Zumft G Cell biology and molecular basis of denitrification Microbiol Mol Biol Rev 1997 61 533 616 9409151 10.1128/mmbr.61.4.533-616.1997 PMC232623

